# Embryonic Development of *Rhynchophorus palmarum* (Coleoptera: Curculionidae): Dynamics of Energy Source Utilization

**DOI:** 10.1093/jisesa/ieu142

**Published:** 2014-01-01

**Authors:** Camilla C. Santana, Josiel S. do Nascimento, Mariana M. Costa, Antonio T. da Silva, Camila B. Dornelas, Luciano A. M. Grillo

**Affiliations:** Pharmacy School, Federal University of Alagoas, Maceió-AL 57072-900, Brazil

**Keywords:** egg, lipid, carbohydrate, embryogenesis, Coleoptera

## Abstract

Energy homeostasis is an essential process during oogenesis, nutrients are required for suitable embryonic development, and recently, studies have investigated metabolic activity during this process. This work aims the investigation of dynamics of energy source utilization of *Rhynchophorus palmarum* during embryogenesis. For this, we first evaluated the mobilization kinetics of the lipids and glycogen. Thereafter, the synthesis of RNA, protein, and the involvement of enzyme of the glycolytic and pentose-phosphate pathways. Results showed that lipid content decreased in contrast with the lipase activity. The total glycogen amounts it was partly consumed and the glucose content increased, but then values remained stable until hatching. Total RNA content increased, and no significant changes in total protein content were observed. A study of the glycolytic pathway data showed activity of hexokinase and pyruvate kinase at the beginning of embryogenesis. Furthermore, glucose-6-phosphate formed is driven into the pentose-phosphate pathway viewed the high activity of glucose-6-phosphate dehydrogenase. Finally, these results showed that mobilization of different energy sources together with different enzymatic activities has an important role in embryonic development of *R. palmarum* .

*Rhynchophorus palmarum* , L. (Coleoptera: Curculionidae) occurs on plant species of 12 different families. Countries reporting the largest damage to crops in palm plantations include Costa Rica, Colombia, Venezuela, and Brazil. *R. palmarum* has been reported as one of the most important pests on commercial palm plantations ( Sánchez and Cerda 1993 ). The larvae feed on the growing tissue in the crown of the palm, often destroying the apical growth area and causing eventual death of the palm. Sánchez et al *.* (1993) studied the biology and the behavior of the insect including courtship, mating, and oviposition in the laboratory. They indicated that the females deposit their eggs into holes in the plant made by the rostrum, normally when the surface of the plant tissue presents some damage, near or on the internodal area of the palm trunk. Eggs are then oviposited individually in randomly distributed holes and rests in a vertical position in the hole, which is sealed by the female with a brown waxy secretion. Female may lay an average of 245 eggs during a period of 30 d and eggs located individually 1–2 mm inside soft plant tissue ( Sánchez et al. 1993 ). Old eggs often show the ondulatory movements of the emerging larvae, which show their darker cephalic coloration through the chorion of the egg.

The energetic metabolism of the developing embryo is tightly regulated, because many biosynthetic pathways which are activated to support the molecular backbone needed for embryonic growth ( Matova and Cooley 2001 ). The high energy demand is supplied by the catabolism of biomolecules such as carbohydrates and lipids, and current literature provides ample data regarding the regulatory cascade of events that control development. In the embryogenesis, the nutrients stored in the form of yolk granules are totally directed to the development of the embryo ( Sappington and Raikhel 1998 ). In the maturation phase, the oocytes exhibit accumulating RNAs, sugars, lipids, and proteins, essential for the development of the embryo ( Chippendale 1978 ). In this process, the lack of nutrients may restrict or even block the developing embryo within the egg ( Thompson and Russel 1999 ). Campos et al. (2006) demonstrated that proteins are the most abundant substrates for embryos during development and that the metabolism of these is directly related to the activity level of carbohydrate metabolism and also to the efficiency of the Krebs cycle. The *Tribolium castaneum* ’s metabolic status during early embryogenesis showed that glucose and glycogen regulation is important for early *T. castaneum* embryonic patterning ( Fraga et al. 2013 ). Given these facts, it is clear that the embryos development has different mechanisms for use of substrates, such as amino acids, lipids, and glucose to control the flow of energy in embryogenesis. Development in invertebrates has been analyzed under different aspects; however, there is a lack of information regarding the dynamics of energetic metabolism during embryogenesis ( Edgar and Lehman 1994 , Abreu et al. 2008 ). This work evaluated some aspects of the kinetics of the utilization of the main potential energy sources available during the embryonic development of the *R. palmarum* . Our results show that lipids, glucose, and glycogen metabolism is important for embryonic patterning. Activity analysis of lipase, hexokinase, pyruvate kinase, and glucose-6-phosphate dehydrogenase suggests important roles of these enzymes during embryogenesis.

## Materials and Methods

### 

#### Insects and Eggs

Insects were taken from a colony of *R. palmarum* maintained at 28°C and 80% relative humidity until the end of oviposition. For embryo development, eggs were maintained under the same conditions and collected at 0, 12, 24, 36, 48, 60, 72, 84, or 96 h after oviposition and stored in a freezer at −20°C.

#### Determination of Glucose Concentration

Eggs were homogenized in 1 ml of phosphate-buffered saline (PBS), pH 7.4, and centrifuged at 10,000 × *g* for 15 min. Glucose was enzymatically quantified in supernatant (100 μl) at different days after oviposition by incubation for 30 min at 37°C, using Glucox ( www.doles.com.br ). The standard mixture contains 3 units/ml of glucose oxidase, 0.3 unit/ml peroxidase, 0.5 mM aminoantipyrine, and 0.5 mM p-hydroxybenzoate. The product formed by the oxidation was read in a Shimadzu (Japan) U1240 spectrophotometer at 510 nm. The absorbance is directly proportional to the concentration of glucose. Three samples were analyzed for each experimental point.

#### Glycogen Content

Eggs were homogenized in 1 ml of extraction buffer containing 200 mM sodium acetate, pH 4.8, and centrifuged at 10,000 × *g* for 10 min. After that, 100 μl (20 mg ptn/ml) of the supernatant was incubated with 1 unit of α-amyloglucosidase (Sigma Chemicals, www.sigmaaldrich.com ) for 4 h at 40°C. Glucose produced was determined as described above. Three samples were analyzed for each experimental point.

#### Determination of Lipids Concentrations

Eggs (15 mg) were homogenized in PBS, pH 7.4, and centrifuged at 10,000 × *g* for 15 min. The supernatant was extracted with chloroform–methanol–water (1:2:0.5; v/v) as described by Bligh and Dyer (1959) . The organic phase was dried under an N _2_ stream and weighed to determine lipid content. Three samples were analyzed for each experimental point.

#### Analysis of Lipase Activity (LP)

Eggs were homogenized in PBS, pH 7.4, and centrifuged at 10,000 × *g* for 15 min. The supernatant was used for analysis of LP, as described elsewhere ( Choi et al. 2003 ). The standard reaction mixture (10 mM 2,3-dimercapto-1-propanol tributyrate [DMPTB], 40 mM 5,5’-dithiobis(2-nitrobenzoic acid) [DTNB], 0.5 M ethylenediaminetetraacetic acid, 10% Triton X-100, and 1 M Tris-Cl, pH 7.5) was mixed in a microcentrifuge tube, and deionized water was added to make a final volume of 900 µl. Microplate wells were filled with 180 µl of this mixture, and 20 µl of the enzyme sample was added to each well. For the specific detection of LP, we used a blank that contained no DMPTB. The microplate was immediately transferred to a 37°C incubator to start the reaction, and the absorbance of each well at 405 nm was measured. In the DMPTB–DTNB method, free thiol groups that are generated by the lipase hydrolysis of DMPTB reduce DTNB to create a yellow color. Three samples were analyzed for each experimental point.

#### RNA Content

For total RNA extraction, 25 mg of egg homogenate was incubated with 600 μl of Trizol reagent ( www.lifetechnologies.com ) at room temperature for 5 min and centrifuged at 14,000 g for 15 min at 4°C. The supernatant was removed, 200 μl of chloroform was added (Merck, Darmstadt, Germany), the sample incubated for 2 min, and centrifuged again. The supernatant was transferred and RNA precipitated with 0.5 ml of isopropanol for 10 min at room temperature, centrifuged, and the pellet resuspended in 1 ml of water. RNA samples were treated with DNase I (Thermo Fisher Scientific, Waltham, MA). Subsequently, absorbance was measured at 260 and 280 nm ( Sambrook et al. 1989 ). Three samples were analyzed for each experimental point.

#### Determination of Protein Concentration

Eggs (10 mg) were homogenized in 1 ml of PBS, pH 7.4, 100 mM leupeptin, 100 nM pepstatin, 1 mM benzamidine, and centrifuged at 10,000 × *g* for 15 min. The supernatant was used for protein concentrations determined using bovine serum albumin as a standard ( Lowry et al. 1951 ). Three samples were analyzed for each experimental point.

#### Hexokinase Activity

Eggs were homogenized in 1 ml of extraction buffer containing 20 mM Tris–HCl, pH 7.5, and centrifuged at 10,000 × *g* for 10 min. The supernatant (40-mg protein/ml) was assayed for HK activity in 20 mM Tris–HCl pH 7.5 containing 6 mM MgCl _2_ , 1 mM ATP, 0.5 mM NAD ^+^ , 10 mM NaF, and 2 mM glucose. The reaction was started by adding protein fraction and was stopped after 5 min at 35°C by heating for 1 min at 100°C. The glucose-6-phosphate formed was measured by adding equal volume of a solution containing 20 mM Tris–HCl pH 7.5, 6 mM MgCl _2_ , 1 unit/ml of glucose-6-phosphate dehydrogenase from *Leuconostoc mesenteroides,* and 0.3 mM β-NAD ^+^ . The production of β-NADH was measured by the increase in absorbance at 340 nm for 5 min (Δ *A*_340_ /min). Three samples were analyzed for each experimental point.

#### Pyruvate Kinase Activity

Eggs were homogenized in 1 ml of extraction buffer containing 20 mM Tris–HCl, pH 7.5, and centrifuged at 10,000 × *g* for 10 min. The supernatant (40-mg protein/ml) was assayed for PK activity in 20 mM Tris–HCl, pH 7.5, 5 mM MgCl _2_ , 1 mM ADP, 0.4 mM NADH, and 1 unit/ml of lactate dehydrogenase, and the reaction was started with 1 mM PEP. The β-NADH consumption was read for 5 min in a Shimadzu U1240 spectrophotometer at 340 nm (Δ *A*_340_ /min) as described by Worthington (1988) . Three samples were analyzed for each experimental point.

#### Glucose-6-Phosphate Dehydrogenase Activity

Eggs were homogenized in 1 ml of extraction buffer containing 55 mM Tris–HCl pH 7.8 and centrifuged at 10,000 × *g* for 10 min. The supernatant was assayed in 150 μl 55 mM Tris–HCl pH 7.8 containing 6 mM MgCl _2_ , 100 mM glucose-6-phosphate, and 0.5 mM β-NADP ^+^ . The reaction was started with 30 μl (40-mg ptn/ml) of homogenate, and β-NADPH formation was monitored at 340 nm during 5 min (Δ *A*_340_ /min) as described by Worthington (1988) . Three samples were analyzed for each experimental point.

#### Statistical Analysis

Data are expressed as the mean ± SD. Statistical comparisons were made by one-way analysis of variance with posttest. Significance levels were 0.05 and 0.01.

## Results

**Fig. 1.**
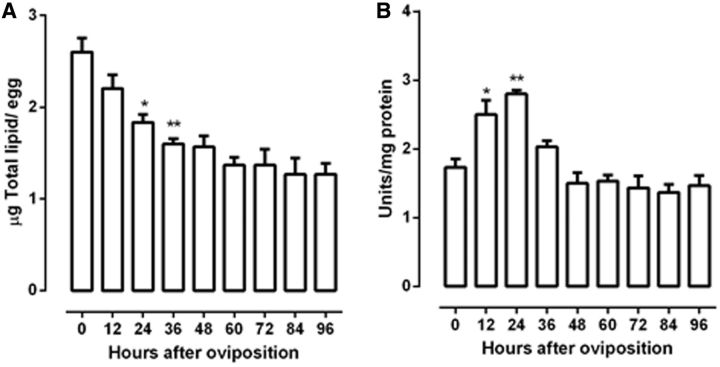
Total lipid amount and LP in *R. palmarum* embryogenesis. Egg homogenate aliquots were subjected to total lipid extraction as described by Bligh and Dyer (1959) . LP was measured with DMPTB and DNTB as described by Choi et al. (2003) . Total lipid amount (A) and LP (B). Results are expressed as mean ± SD for three independent determinations. * *P* < 0. 05 versus time 0; ** *P* < 0.01 versus time 0.

This work evaluated the mobilization kinetics of some components of *R. palmarum* during embryogenesis. We first established the time course of the lipid mobilization and LP. Results showed that lipid content decreased during embryogenesis, dropping to 1.8 μg/egg at 24 h ( *P* < 0.05) and 1.5 μg at 36 h ( *P* < 0.01) ( Fig. 1 A). Between 48 and 96 h after oviposition, lipid content further decreased and was statically different of the amount present in the initial; however, there is no significant change between them. LP increased in the first 12 h ( *P* < 0.05) reached at maximum activity at 24 h ( *P* < 0.01) after egg laying and decreased together with the lipid content ( Fig. 1 B).

**Fig. 2.**
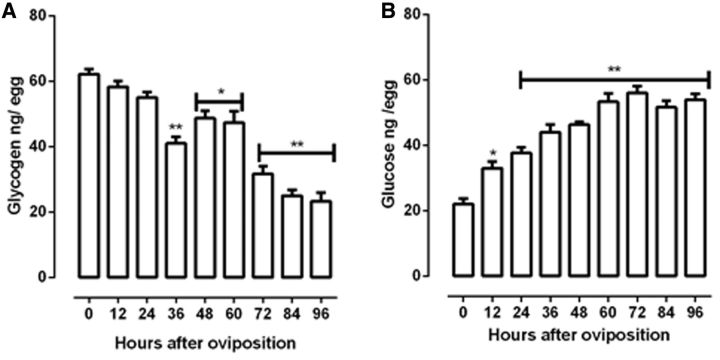
Glycogen and glucose content in *R. palmarum* embryogenesis. The glycogen (A) amount was determined by digestion with α-amyloglucosidase, and glucose (B) released was measured as described in Materials and Methods. Results are expressed as mean ± SD for three independent determinations. * *P* < 0.05 versus time 0; ** *P* < 0.01 versus time 0.

The total glycogen amounts in the eggs showed that it was partly consumed until 36 h, about 30% relative at initial of embryogenesis ( Fig. 2 A). Afterward, then showed a little increase between 48 and 60 h and was drastically decrease until 96 h of embryonic development ( *P* < 0.05 and *P* < 0.01). In contrast, the glucose content was statically different at 12 h after oviposition ( *P* < 0.05), increased until 60 h, and values remained stable until hatching ( *P* < 0.01) ( Fig. 2 B).

**Fig. 3.**
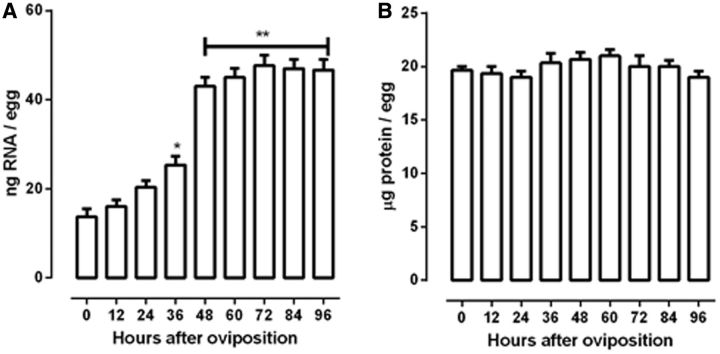
Total protein and RNA content in *R. palmarum* embryogenesis. Total RNA (A) was extracted with Trizol reagent (Life Technologies) according to the manufacturer’s instructions. Total protein (B) was measured using Lowry method. Results are expressed as mean ± SD for three independent determinations. * *P* < 0. 05 versus time 0; ** *P* < 0.01 versus time 0.

The changes in total RNA and protein amount during embryogenesis showed that total amount of RNA was statistically different at 36 h ( *P* < 0.05), then rose quickly until 48 h ( *P* < 0.01) and did not exhibit significant changes till the end of embryogenesis ( Fig. 3 A). No significant changes in total protein content were observed throughout *R. palmarum* embryogenesis ( Fig. 3 B).

**Fig. 4.**
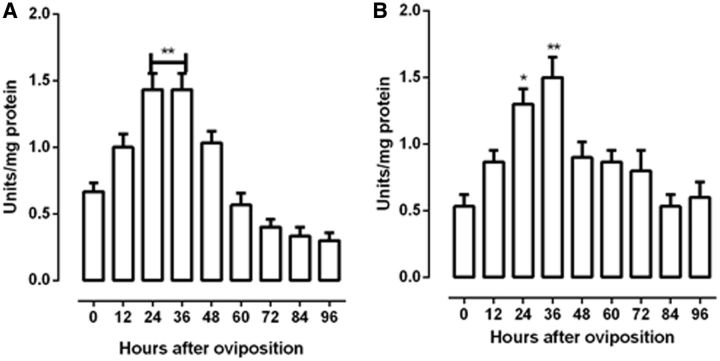
Hexokinase and pyruvate kinase activities during *R. palmarum* embryogenesis. The hexokinase (A) and pyruvate kinase (B) activities were measured in egg homogenates on different days of embryo development. The β-NADH production or consumption was monitored at 340 nm. The activities were assayed as described in Materials and Methods. Results are expressed as mean ± SD for three independent determinations. * *P* < 0.05 versus time 0; ** *P* < 0.01 versus time 0.

The study of the glycolytic pathway was evaluated from the measurements of the activity of hexokinase and pyruvate kinase. Hexokinase activity rapidly increased at the 24 and 36 h and showed statistically different ( *P* < 0.01), compared with the initial of embryogenesis. At 48 h after oviposition, hexokinase activity starts to decrease until 96 h ( Fig. 4 A). Pyruvate kinase activity correlated with hexokinase, because it was high at 24 h ( *P* < 0.05), increased at the maximum level at 36 h ( *P* < 0.01), and decreased thereafter until values of initial of embryogenesis ( Fig. 4 B).

**Fig. 5.**
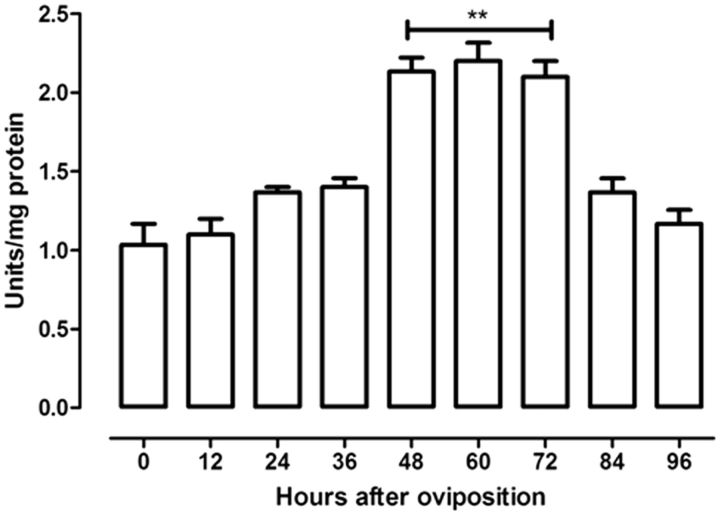
Glucose-6-phosphate dehydrogenase activity during *R. palmarum* embryogenesis. The glucose-6-phosphate dehydrogenase activity was measured on different days of embryo development. The β-NADH production was monitored at 340 nm. The activities were assayed as described in Materials and Methods. Results are expressed as mean ± SD for three independent determinations. * *P* < 0. 05 versus time 0; ** *P* < 0.01 versus time 0.

The pentose-phosphate pathway activity during embryogenesis was investigated by the measurement of the glucose-6-phosphate dehydrogenase profile activity, which is the rate-limiting step in this route. There is a relatively high glucose-6-phosphate dehydrogenase activity that reached a peak between 48 and 72 h after oviposition. Then, the activity rapidly decreased and remained at low levels until the end of embryogenesis ( Fig. 5 ).

## Discussion

Mobilization of egg components has also been the subject of several reports ( Abreu et al. 2008 ; Santos et al. 2008 , 2011 ). This work evaluated the kinetics from the utilization of the main potential energy sources available during the embryonic development of the *R. palmarum* . Quantification of the major constituents of the egg in the embryogenesis course suggests that lipids and carbohydrates are the major energy sources for the quick embryo segmentation in different arthropod models ( Nusslein-Volhard and Roth 1989 , Bate and Arias 1993 ).

The total lipid content amount decreased during embryogenesis, showing that it was consumed upon the end of embryogenesis ( Fig. 1 A). Simultaneously, the LP increase in the initial of the embryogenesis until the 24 h and decrease together with the lipids ( Fig. 1 B). Therefore, it seems that lipids are used as fuel, and the LP seems to be important for lipid degradation. These was similar to that of eggs of the kissing bug *Rhodnius prolixus* , the butterﬂy *Bicyclus anynana* , and *B. microplus* ( Campos et al. 2006 , Geister et al. 2009 , Santos et al. 2011 ).

In *R. palmarum* , the glucose content increased and remain stable until hatching, but the glycogen amounts is consumed ( Fig. 2 A and B). These results agree with what is described in literature. Previous reports have noted that glycogen is consumed as the embryo develops ( Regano-Caracciolo et al. 1998 , Santos et al. 2008 ). The utilization of carbohydrates in the course of development of the embryo was described in cattle tick *B. microplus* ( Campos et al. 2006 ). A similar result was observed in the camel tick *Hyalomma dromedarii* embryogenesis ( Mohamed 2000 ).

An increase in total RNA content at the beginning of embryonic development has also been described for *Tribolium confusum* and for *B.microplus* ( Amnai-Devi et al. 1963 , Campos et al. 2006 ). The increase in total RNA content after oviposition in *R. palmarum* probably reflects also an intense zygotic transcription activity ( Fig. 3 A). Changes in RNA content reflect the anabolic activities of the developing embryo and the degree of embryonic independence of the material of maternal origin. Proteins are the largest components of the yolk, and the total protein content in *R. palmarum* eggs was unchanged from oviposition to hatching ( Fig. 3 B). This is in accordance with data from *H. dromedarii* and *B. microplus* ( Kamel et al. 1982 , Campos et al. 2006 ). The results show that the mobilization of yolk reserves represents the main metabolic strategy adopted by these insects to meet their biosynthetic demands during embryogenesis.

In different marine species, it was observed that there are significant increases in carbohydrates metabolism during embryogenesis, especially glycolysis ( Thompson 2000 , Lahnsteiner and Patarnello 2003 , 2004 , Lahnsteiner 2005 ). Fraga et al. (2013) showed high levels of hexokinase activity in the first 4 h of development and a decrease to about one third of the initial activity in the 4–8 h of embryonic development on *T. castaneum* . In *B. microplus* , the hexokinase activity is relatively high on the first 3 d of embryogenesis, and the pyruvate kinase activity does not correlate with hexokinase activity in the beginning of embryogenesis ( Moraes et al. 2007 ).

In *R. palmarum,* the levels of hexokinase activity increase between 24 and 36 h after oviposition ( Fig. 4 A). Hexokinase can phosphorylate glucose originated from glycogen breakdown. This is in agreement with data obtained in *T. castaneum* and *B. microplus* ( Moraes et al. 2007 , Fraga et al. 2013 ). In contrast to Moraes et al. (2007) , we found that pyruvate kinase activity increases in the beginning of embryogenesis and follow the decrease of hexokinase activity ( Fig. 4 B).

Glucose-6-phosphate dehydrogenase activity reached a peak between 48 and 72 h after oviposition ( Fig. 5 ). The high activity can be explained by the intense RNA formation that occurs in this period. The pentose-phosphate pathway may be supplying the nucleotide synthesis with ribose-5-phosphate and NADPH ( Moraes et al. 2007 ). A part of the glucose-6-phosphate produced may be not used in glycolysis, as there is a significant decrease in pyruvate kinase activity ( Fig. 4 B), simultaneously to increase in glucose 6-phosphate dehydrogenase activity ( Fig. 5 ). Glucose-6-phosphate would drive the pentose-phosphate pathway or would mainly be used for glycolysis. This possibility is supported by two results: 1) the decrease of the hexokinase activity and 2) that its activity precedes the glucose 6-phosphate dehydrogenase activity peak between 48 and 72 h. This indicates that glucose 6-phosphate produced by hexokinase would be used by glucose 6-phosphate dehydrogenase.

In conclusion, energy source are mobilization in response to energy demands for the developing embryo and an increased of synthesis of RNA reflect the anabolic activities in this period. LP seems to be important for lipid degradation, enzymes of the glycolytic and pentose-phosphate pathways are the involvement in the utilization of the glucose and the intense RNA formation. This study is the first description of energy source utilization in the embryonic development of *R. palmarum.* It is an initial step toward further investigations on the pathways and regulatory mechanisms that are involved in these processes.
